# Sparse Representation for Infrared Dim Target Detection via a Discriminative Over-Complete Dictionary Learned Online

**DOI:** 10.3390/s140609451

**Published:** 2014-05-27

**Authors:** Zheng-Zhou Li, Jing Chen, Qian Hou, Hong-Xia Fu, Zhen Dai, Gang Jin, Ru-Zhang Li, Chang-Ju Liu

**Affiliations:** 1 College of Communication Engineering, Chongqing University, Chongqing 400044, China; E-Mails: chenjingsunshing@gmail.com (J.C.); shuijingxin@gmail.com (Q.H.); fhxma092@gmail.com (H.-X.F.); daizhenlite@gmail.com (Z.D.); 2 National Laboratory of Analogue Integrated Circuits, No. 24 Research Institute of China Electronics Technology Group Corporation, Chongqing 400060, China; E-Mails: lrzmail@163.com (R.-Z.L.); liucj@cetccq.com.cn (C.-J.L.); 3 China Aerodynamics Research and Development Center, Mianyang 621000, China; E-Mail: gjin@ioe.ac.cn

**Keywords:** dim target detection, adaptive morphological over-compete dictionary, discriminative over-complete dictionary, Gaussian over-complete dictionary, sparsity

## Abstract

It is difficult for structural over-complete dictionaries such as the Gabor function and discriminative over-complete dictionary, which are learned offline and classified manually, to represent natural images with the goal of ideal sparseness and to enhance the difference between background clutter and target signals. This paper proposes an infrared dim target detection approach based on sparse representation on a discriminative over-complete dictionary. An adaptive morphological over-complete dictionary is trained and constructed online according to the content of infrared image by K-singular value decomposition (K-SVD) algorithm. Then the adaptive morphological over-complete dictionary is divided automatically into a target over-complete dictionary describing target signals, and a background over-complete dictionary embedding background by the criteria that the atoms in the target over-complete dictionary could be decomposed more sparsely based on a Gaussian over-complete dictionary than the one in the background over-complete dictionary. This discriminative over-complete dictionary can not only capture significant features of background clutter and dim targets better than a structural over-complete dictionary, but also strengthens the sparse feature difference between background and target more efficiently than a discriminative over-complete dictionary learned offline and classified manually. The target and background clutter can be sparsely decomposed over their corresponding over-complete dictionaries, yet couldn't be sparsely decomposed based on their opposite over-complete dictionary, so their residuals after reconstruction by the prescribed number of target and background atoms differ very visibly. Some experiments are included and the results show that this proposed approach could not only improve the sparsity more efficiently, but also enhance the performance of small target detection more effectively.

## 1. Introduction

The distance between man-made satellites and ground-based EO sensors is usually more than 30,000 km, and the angle between the object and ground-based EO sensor is so small that the target on a EO sensor is a small blob with only several pixels. Meanwhile, the energy of the object decays greatly for long distance propagation, and it is usually submerged in noise and clutter. This causes great difficulty for infrared dim small target detection and tracking [1,2]. The problem of how to effectively distinguish dim small targets from clutter has been widely studied over the past years, and a number of dim target detection algorithms have been developed and they can approximately be classified into two categories, namely, detection before track (DBT) and track before detection (TBD) [3–5]. Image filtering and content learning are the two basic methods of DBT-based target detection algorithms. The image filtering-based detection algorithms such as Top-Hat [6], TDLMS [7] and wavelet transform [8,9] usually whiten the image signal, and then determine whether there is a target or not in every scan by the amplitude threshold using some criteria, such as constant false alarm ratio (CFAR). The content learning-based target detection algorithms such as principal component analysis (PCA) compare the similarity of the image and the template pre-learned by knowledge [10]. The TBD-based algorithms jointly process more consecutive scans and declare the presence of a target and its track by searching the candidate trajectory using an exhaustive hypothesis. Temporal cross product (TCP) is presented to extract the characteristics of temporal pixels by using a temporal profile in infrared image sequences, and it could effectively enhance the signal-to-clutter ratio (SCR) [11]. Higher detection probability and lower false alarm probability in every scan could not only facilitate analysis of characteristics, including movement analysis, but also simplify the computational complexity for TBD.

The sparse representation decomposed on an over-complete dictionary is a newly-developed content learning-based target detection algorithm strategy [12–14]. In the over-complete dictionary, there are large number of atoms representing target and even background [15]. Gaussian [16,17] and Gabor [18] are the representative functions used to construct structural over-complete dictionaries offline. It is difficult for these structural over-complete dictionaries to suit the target and background with non-structure shape, so their representation coefficient vectors would be not sparse enough to distinguish targets from background clutter [19]. The adaptive morphological component over-complete dictionary is constructed according to the image content, and it could enhance the sparsity of the representation coefficient vector. However, the atoms representing target and background are mixed together, and the sparsity of the representation coefficient vector is usually too lower to detect target signals [20], therefore, it is necessary to discriminate the atoms representing targets from the ones describing background clutter. The existing techniques to discriminate atoms usually choose background clutter to train a background over-complete dictionary, and manually select target signals to build a target over-complete dictionary. The discriminative over-complete dictionary trained manually could greatly improve the sparsity of the representation coefficient vector and also improve the performance of dim target detection. Nevertheless, its serious limitation is that the atoms couldn't adapt to moving targets and changing backgrounds effectively, so it is necessary to distinguish the atoms automatically for the discriminative over-complete dictionary in order to further enhance the capability of dim target detection.

An infrared dim target detection approach based on sparse representation over discriminative over-complete dictionary learned online is proposed in this paper. An adaptive morphological over-complete dictionary is built according to infrared image content by a K-singular value decomposition (K-SVD) algorithm [21], and then a target over-complete dictionary is discriminated automatically from a background over-complete dictionary by the criteria that the atoms representing dim target signals could be decomposed more sparsely over a Gaussian over-complete dictionary than the one in a background over-complete dictionary. The remainder of the paper is organized as follows: the adaptive over-complete dictionary is trained in Section 2. It is further divided into target over-complete dictionary and background over-complete dictionary in Section 3. The sparsity-driven dim target detection approach is presented in Section 4. Some experiments are included in Section 5 to evaluate the performance of the discriminative over-complete dictionary and Gaussian over-complete dictionary, and the results show that the target detection performance achievable by the proposed approach is significantly enhanced. Conclusions are drawn in Section 6.

### 2. Adaptive Morphological Component Dictionary

Infrared dim target images consists of target, background and noise, and can be modeled as [5]:

(1) $$\{\begin{array}{lll} H_{1}: & f=f_{t}+f_{b}+n & \text{target present}\\ H_{0}: & f=f_{b}+n & \text{target absent} \end{array}$$

where *f* is the infrared image, *f_t_*, *f_b_*, and *n* are the target, background and noise, respectively. Dim target detection is a two-category classification problem, *i.e.,* pixels are labeled as target (target present) or background (target absent) based on their diverse characteristic differences. The dim target concentrates itself relatively in a small other than pixel-sized object region with uniform amplitude, and could be described by point spread function:

(2) $$f_{t}\;x,y,k=a\;k\cdot \exp\left\{-\frac{1}{2}\left[{\left(\frac{x-x_{t}\;k}{\delta_{x}\;k}\right)}^{2}+{\left(\frac{y-y_{t}\;k}{\delta_{y}\;k}\right)}^{2}\right]\right\}$$

where, *a k* is target intensity amplitude; *x_t_*, *y_t_* denotes the target location at instant *k*, and *x_t_* and *y_t_* represent the horizontal and vertical direction, respectively; *δ_x_ k* and *δ_y_ k* are the extent parameters at horizontal and vertical direction, respectively, and they are usually several pixels for their angle is very small when the distance between the man-made satellite and ground-based EO receiver is 30,000 km. Moreover, the signal-to-noise (SNR) is always also low because the target's energy decays greatly when it propagates in noise and clutter.

The K-singular value decomposition (K-SVD) algorithm is adopted to learn the image content and train adaptive over-complete dictionary **D** from a large number of infrared dim images. The dictionary is trained by the following formula [22]:

(3) $$\gamma ,\text{D}=\underset{\text{D},\gamma }{\text{argmin}}\;\text{∑}{\left\|\gamma \right\|}_{0}+\text{∑}{\left\|\text{D}\gamma -f\right\|}_{2}^{2}$$

where ∥·∥_0_ denotes ℓ_0_ − norm , which is defined as the number of nonzero entries in the vector, and ∥·∥_2_ denotes ℓ_2_ − norm , which is defined as the energy of the vector. The formula means the infrared image *f* could be decomposed on the over-complete dictionary **D**, and the representing coefficient vector **γ** has the least number of nonzero entries under the constraint that the residual ${\left\|f-\text{D}\gamma \right\|}_{2}^{2}$ is the minimum. Obviously, the sparsity of the representation coefficients would be dominated by the over-complete dictionary **D.**

The two terms **D** and **γ** should be solved simultaneously in Equation (3). The construction of the adaptive over-complete dictionary is an iterative process, and there are two stages in every iteration, namely, sparse coding and dictionary update [23]:

(1) Sparse coding. In this stage, assuming that **D** is fixed; the sparse representation **γ** is updated by solving the following formula:
(4) $$\mathrm{argmin}{\left\|\gamma \right\|}_{0}s.t.{\left\|f-\text{D}\gamma \right\|}_{2}^{2}\leq \varepsilon$$
  It means that the sparsest representation vector **γ** is searched by an orthogonal matching pursuit (OMP) algorithm under the constraint that the residual ${\left\|f-\text{D}\gamma \right\|}_{2}^{2}$ would be less than the error tolerance ***ε*** [24,25].
(2) Dictionary **D** update. During this stage, only one atom *d_k_* in the dictionary **D** is updated at every iteration, and then the residual is estimated by:
(5) $$E_{\text{k}}={\left\|f-\underset{k}{\text{∑}}d_{k}\gamma_{k}\right\|}_{2}^{2}$$

Therefore, an approximate solution set (*d_k_*,*γ_k_*) would be optimized by the SVD algorithm. Repeating Equation (5), every atom *d_k_* in the dictionary **D** will be updated until the residual $\left\|f-\underset{k}{\text{∑}}d_{k}\gamma_{k}\right\|$ is less than the error tolerance ***ε***. The signal representation error decays exponentially with increasing iteration number, and the adaptive dictionary would be constructed after a few iterations. The final version dictionary **D**, which called adaptive morphological over-complete dictionary, would be compatible with the content of infrared image.

**Figure 1.**
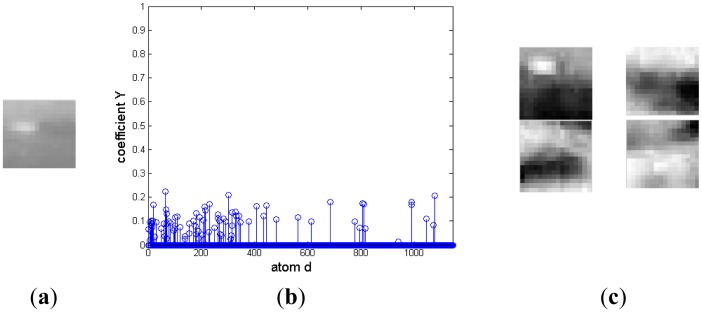
Decomposition of a target image block on adaptive morphology over-complete dictionary. (**a**) Target image block. (**b**) Representation coefficient. (**c**) Four atoms with maximum coefficients.

In the morphological over-complete dictionary **D**, the atoms representing target and background are mixed together. This could induce two difficulties for the signal is decomposed based on not only the target over-complete dictionary, but also the background over-complete dictionary. One is that the representing coefficients mightn't be sparse. The other is that the representing coefficients are irregular and couldn't easily discriminate target from background. To better illustrate the challenge, an example is shown in Figure 1. There is a target in Figure 1a. Its representation coefficient decomposed on the over-complete dictionary **D** is shown in Figure 1b. The dictionary contains 1,144 atoms. There are many nonzero coefficients on atoms representing target and background, and the four atoms corresponding to the largest four nonzero entries are shown in Figure 1c. The first atom represents the target, and the other three atoms describe the background. Therefore, it is necessary to discriminate the atoms representing the target from the ones describing background clutter.

### 3. Discriminative Over-Complete Dictionary Constructed Online

The sparse representation model assumes that the signal could be reconstructed with the same type of over-complete dictionary and corresponding representation coefficients [26]. For the background signal *f_b_*, it can be represented by a linear combination of the background atoms:

(6) $$\begin{array}{ll} f_{b} & \approx \alpha_{1}d_{1}^{b}+\alpha_{2}d_{2}^{b}+\ldots +\alpha_{N_{b}}d_{N_{b}}^{b}\\ & =\left[d_{1}^{b},d_{2}^{b},\ldots ,d_{N_{b}}^{b}\right]\;{\left[\alpha_{1},\alpha_{2},\ldots ,\alpha_{N_{b}}\right]}^{T}={\text{D}}_{\text{b}}\alpha \end{array}$$

where **D_b_** is the background over-complete dictionary, *N_b_* denotes the account of atoms in the dictionary **D_b_**, and **α** is the representing coefficient vector whose entries are the abundances of the corresponding atoms in **D_b_**, *i.e.,* sparse vector or a vector with only few nonzero entries.

Similarly, for the target signal *f_t_*, it can also be sparsely represented as a linear combination of the target atoms:

(7) $$\begin{array}{ll} f_{t} & \approx \beta_{1}d_{1}^{t}+\beta_{2}d_{2}^{t}+\ldots +\beta_{N_{t}}d_{N_{t}}^{t}\\ & =\left[d_{1}^{t},d_{2}^{t},\ldots ,d_{N_{t}}^{t}\right]\;{\left[\beta_{1},\beta_{2},\ldots ,\beta_{N_{t}}\right]}^{T}={\text{D}}_{\text{t}}\text{β} \end{array}$$

where **D_t_** is the target over-complete dictionary, *N_t_* is the number of atoms in the target dictionary **D_t_** and **β** is the sparse representing coefficient vector. An infrared image lies in the union of the background dictionary and target dictionary, *i.e.,*

(8) $$f={\text{D}}_{\text{b}}\alpha +{\text{D}}_{\text{t}}\beta =\underset{\text{D}}{\underset{︸}{\begin{array}{cc} {\text{D}}_{\text{b}} & {\text{D}}_{\text{t}} \end{array}}}\underset{\gamma }{\left[\begin{array}{c} \alpha \\ \beta \end{array}\right]}=\text{D}\gamma$$

where **D** = [**D_b_ D_t_**] is a matrix consisting of both background and target atoms, and **γ** = [**α β**] is a (*Nt* + *N_b_*) – dimentional vector consisting of the two vectors **α** and **β** associated with the two dictionaries.

Since background clutter and the target signal usually consist of different materials, they have distinct signatures and thus lie in different over-complete dictionaries. If an infrared image is a target signal, it ideally can be represented by a target dictionary, but can't be represented by the background atoms. In this case, **α** is a zero vector and **β** is a sparse vector. Therefore, the infrared image can be sparsely represented by the union of background over-complete dictionary and target over-complete dictionary, and the location of nonzero entries in the sparse vector **γ** actually contains critical information about the class of the infrared image.

The existing techniques to discriminate atoms usually choose background clutter to train a background over-complete dictionary, and manually select target signals to build a target over-complete dictionary offline. Nevertheless, the serious limitation of such an offline discriminative over-complete dictionary is that the atoms couldn't effectively adapt to moving targets and changing backgrounds, which would induce the discrimination between target and clutter to be too small to distinguish targets from background clutter, so it is necessary to distinguish automatically the atoms online for the discriminative over-complete dictionary in order to further enhance the capability of dim target detection.

As the Introduction discusses, the distance between a geosynchronous satellite and a ground-based EO receiver is more than 30,000 km, and the angle is so small that the target on the EO sensor is a small blob with only several pixels with Gaussian distribution, so dim small targets usually are described by a two-dimensional Gaussian intensity model (GIM), which is widely used to describe infrared dim small targets:

(9) $$I\;i,j=I_{\max}\exp\left(-\frac{1}{2}\left[\frac{i-{i_{0}}^{2}}{\sigma_{x}^{2}}+\frac{j-{j_{0}}^{2}}{\sigma_{y}^{2}}\right]\right)$$

where (*i*_0_,*j*_0_) is the the center of the blob, *I*(*i*,*j*) is the intensity at the position (*i*,*j*), *I*_max_ is the intensity of the peak, $\sigma_{x}^{2}$ and $\sigma_{y}^{2}$ are the extent parameters at horizontal and vertical directions, respectively.

**Figure 2.**
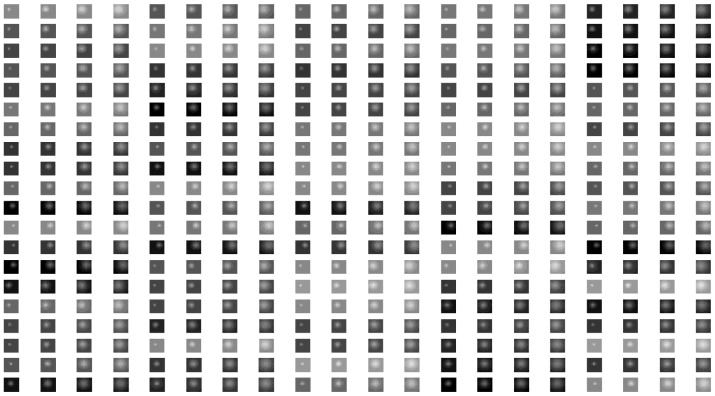
Gaussian over-complete dictionary.

The extent parameters control the target intensity spread degree, and it represents a small point when they are very little, and denote a flat block when they are very large. In content learning-based target detection algorithms, the correlation between infrared image and GIM is usually measured to decide whether there is dim target or not. In this paper, the GIM-based structural over-complete dictionary is adopted to test the atoms of adaptive morphological component dictionary, and automatically discriminate the atoms representing target from the ones describing background clutter. There are four parameters in GIM, namely, center position (*i*_0_,*j*_0_), peak intensity *I*_max_, horizontal and vertical extent parameters $\sigma_{x}^{2}$ and $\sigma_{y}^{2}$, and they are adjusted to generate a large number of diverse atoms with different position, brightness and shape. Figure 2 is a part of the atoms of Gaussian over-complete dictionary **D***_gaussian_*, and each atom has 7 × 7 pixels.

**Figure 3.**
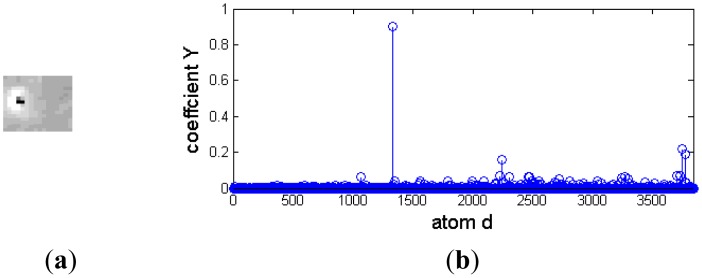

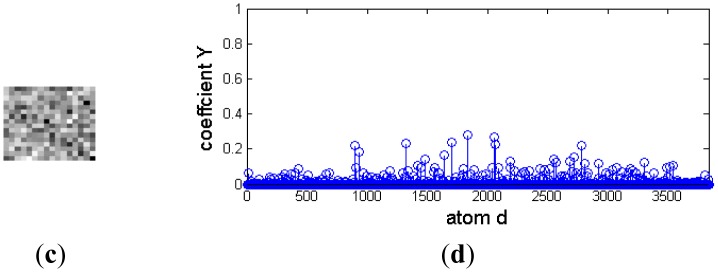
Representation coefficient of a deep space image decomposed based on a Gaussian over-complete dictionary. (**a**) and (**b**) are target signal and its sparse coefficient, respectively; (**c**) and (**d**) are background noise and its sparse coefficient, respectively.

Although a dim moving target is always polluted by environmental noise, it approximately affords a two-dimensional Gaussian model. Otherwise, different background clutter has diverse and abundant morphology. For example, cirrocumulus cloud is composed of small spherical clouds, which arrange in rows or groups; stratocumulus cloud is generally larger and looser, and its thickness and shape are also different. Figure 3 is the representation coefficients of target signal and background noise decomposed based on a Gaussian over-complete dictionary. The target signals and background noise are from a deep space image. Figure 3a and 3b are the target image block and its representation coefficients, respectively. The target signal is a bright blob with a black noise at its center, and it is very similar to a two-dimensional Gaussian function. There are only several nonzero representation coefficients, and the target signal could be decomposed sparsely by a Gaussian over-complete dictionary. Figure 3c and 3d are background noise and its representation coefficients, respectively. Much of its representation coefficients are nonzero, and the background noise should be reconstructed by many Gaussian atoms.

**Figure 4.**
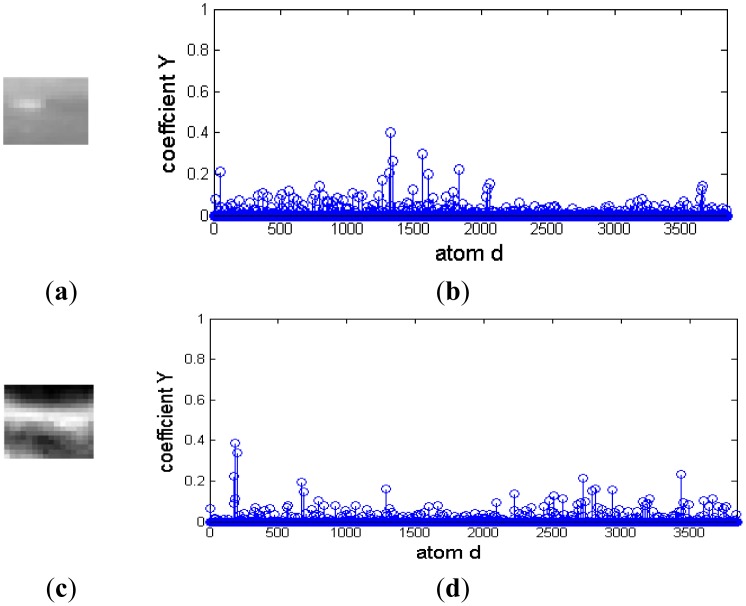
Representation coefficient of cloud image on Gaussian over-complete dictionary. (**a**) and (**b**) are target signal and it sparse coefficient, respectively. (**c**) and (**d**) are cloud background and its sparse coefficient, respectively.

A small infrared target in a cloud background and their representation coefficients decomposed based on a Gaussian over-complete dictionary are shown in Figure 4. Figure 4a and 4b are the target signal and its representation coefficients, respectively. The target signal is a bright rectangle, and it has more nonzero representation coefficients than that of the target signal similar to the two-dimensional Gaussian function shown in Figure 3. Therefore, a target signal like a bright rectangle could be reconstrcted by a few of Gaussian atoms with maximum nonzero representation coefficients. Figure 4c and 4d are the cloud background and its representation coefficients, respectively. It is shown that much of these coefficients are nonzero, and it couldn't be decomposed sparsely. Moreover, the representation coefficient difference between target signal and background is so small that it is hard to distinguish the target from background clutter.

Therefore, compared with the background atom in the adaptive morphological component dictionary, the target atom could be reconstructed from a lesser amount of Gaussian atoms. In other words, with the same number of Gaussian atoms with maximum sparse coefficients, the residual of a background atom would be much greater than that of a target atom. Based on this idea, the atom in the adaptive morphology over-complete dictionary **D** could be classified as target over-complete dictionary **D**_t_ and background over-complete dictionary **D**_b_. The atom *d_k_* is decomposed on Gaussian over-complete dictionary **D***_gaussian_* as follows:

(10) $$\hat{\alpha }=\underset{\alpha }{\arg\min}{\left\|d_{k}-{\text{D}}_{\text{gaussian}}\alpha \right\|}_{2}^{2}s.t.{\left\|\alpha \right\|}_{0}=k$$

where *d_k_* is an atom of **D**. After decomposing for the fixed *k* times, the residual *r*(*d_k_*) of the atom *d_k_* is defined as:

(11) $$r\;d_{k}={\left\|d_{k}-{\text{D}}_{\text{gaussian}}\text{α}\right\|}_{2}$$

Whether the atom *d_k_* is target atom or not could be decided by comparing the residual *r*(*d_k_*) with a threshold *δ*:

(12) $$\{\begin{array}{cc} d_{k}\in {\text{D}}_{\text{t}} & r\;d_{k}\leq \delta \\ d_{k}\in {\text{D}}_{\text{b}} & r\;d_{k}>\delta \end{array}$$

**Figure 5.**
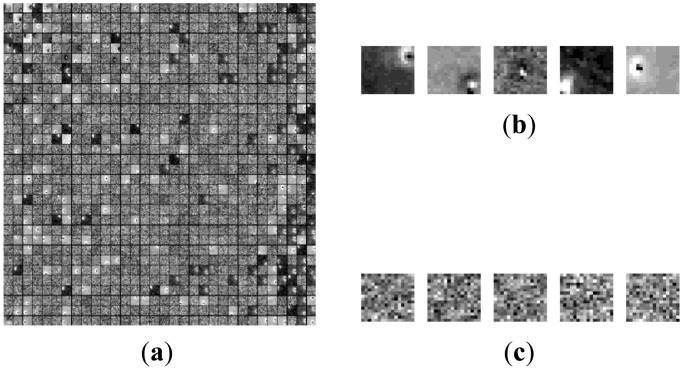
Discriminative over-complete dictionary for space image. (**a**) Dictionary **D**, (**b**) Target atom of **D**_t_, (**c**) Background atom of **D**_b_.

The threshold *δ* usually is proportional to the size of the atom. Every atom of **D** could be identified, then the adaptive background over-complete dictionary **D**_b_ and target over-complete dictionary **D**_t_ could be constructed online and automatically. Figure 5 is an example of the discriminative over-complete dictionary learned online. Figure 5a is a part of adaptive morphological dictionary **D** for space target image. Figure 5b shows five target atoms. The target atoms have abundant shapes, and they can more really reflect the morphological characteristics of the original dim target than Gaussian atom. Five background atoms are listed in Figure 5c, and all of them are noise.

### 4. Dim Target Detection Criteria

Once the target and background over-complete dictionaries **D***_t_* and **D***_b_* are learned and constructed online through these above procedures, the image *f* could be decomposed on these two dictionaries with the error tolerance *σ*, respectively:

(13) $$\hat{\beta }=\mathrm{argmin}{\left\|\beta \right\|}_{0}s.t.{\left\|{\text{D}}_{t}\beta -f\right\|}_{2}\leq \sigma$$

(14) $$\hat{\alpha }=\mathrm{argmin}{\left\|\alpha \right\|}_{0}s.t.{\left\|{\text{D}}_{b}\alpha -f\right\|}_{2}\leq \sigma$$

These formulas are approximately solved by greedy pursuit algorithms such as orthogonal matching pursuit (OMP) or subspace pursuit (SP). The target can be sparsely decomposed over the target over-complete dictionary, yet it can't be sparsely decomposed on the background over-complete dictionary. For target signals, the residual reconstructed by target atoms with maximum representation coefficients would be less than that reconstructed by the same number of background atoms with maximum representation coefficients. Let us define the residual reconstructed by target over-complete dictionary as *r_t_ f*:

(15) $$r_{t}\;f=\sqrt{\overset{m}{\underset{i=1}{\text{∑}}}\left\|f-d_{i}^{t}\beta_{i}\right\|}$$

where *β_i_* denotes the recovered sparse coefficient for *f* associated with the target over-complete dictionary, and *m* is a prescribed constant, such as five. Similarly, background clutter can be sparsely decomposed on the background over-complete dictionary, yet it can't be sparsely decomposed on the target over-complete dictionary. For background clutter, the residual reconstructed by target atoms with maximum representation coefficients would be larger than that reconstructed by the same number of background atoms with maximum representation coefficients. Let us define the residual reconstructed by background over-complete dictionary as *r_b_ f*:

(16) $$r_{b}\;f=\sqrt{\overset{m}{\underset{i=1}{\text{∑}}}\left\|f-d_{i}^{b}a_{i}\right\|}$$

where **α***_i_* denotes the recovered sparse coefficient for *f* associated with the background over-complete dictionary.

The sparsity-based dim target detector can be done by comparing the difference of residuals with a prescribed threshold, *i.e.,*

(17) $$D\;f=r_{b}\;f-r_{t}\;f$$

If *D*(*f*) > *η*, *f* would be labeled as target; otherwise, it would be labeled as background clutter. *η* is a prescribed threshold.

### 5. Experimental Results and Analysis

**Figure 6.**
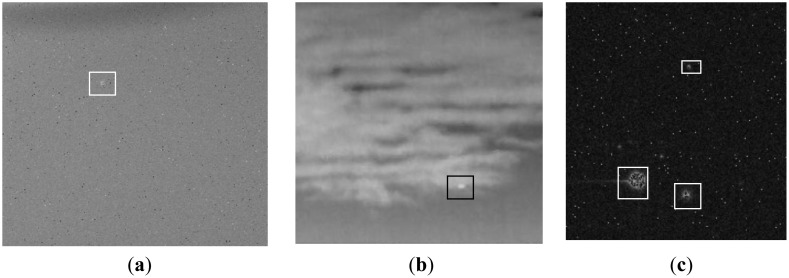
Original infrared image. (**a**) Deep space image. (**b**) Cloud image. (**c**) Multi-target image.

The following experiments have been implemented in MATLAB language on personal computer with a Pentium dual-core CPU E5900. Figure 6 shows the low contrast infrared images, which are captured outfield by an EO imaging tracking system. Figure 6a is the deep space sequence image, Figure 6b is the cloud sequence image, and Figure 6c is the multi-target image. Noise and cloud are the background clutter of deep space images, multi-target images and cloud images, respectively. The target is the brighter maculous form at the center of rectangle box. Their signal-to-noises (SNRs) are about 2.3 and 3.5 in Figure 6a and 6b, respectively. In Figure 6c, three targets with different scale are marked, and their SNRs are different.

**Figure 7.**
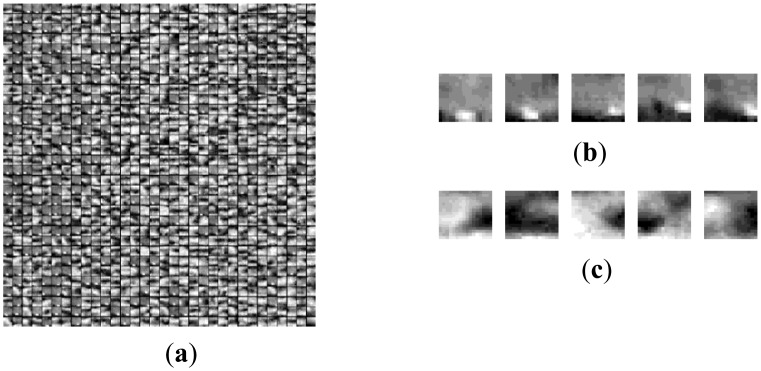
Adaptive over-complete dictionary for cloud image. (**a**) Dictionary. (**b**) Target atom. (**c**) Background atom.

**Figure 8.**
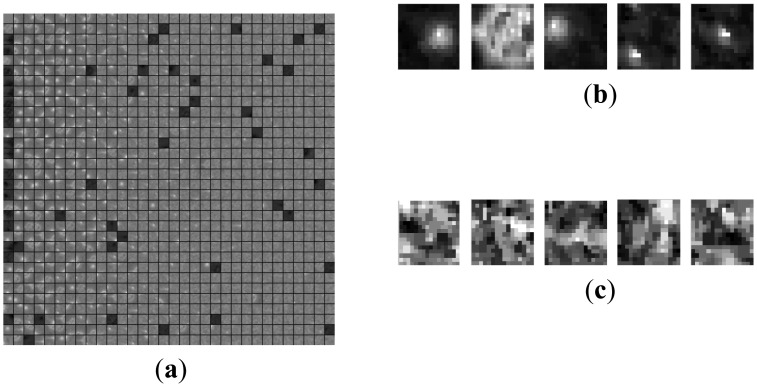
Adaptive over-complete dictionary for Multi-target image. (**a**) Dictionary. (**b**) Target atom. (**c**) Background atom.

Figure 5, 7 and 8 are the morphological over-complete dictionary for the deep space images, cloud images and multi-target images, respectively. Compared with the Gaussian over-complete dictionary shown in Figure 2, the adaptive morphological over-complete dictionary has more diverse and abundant morphology, and it would be more suitable to represent original images with less atoms. Every atom is 7 × 7 pixels.

The space image and cloud image are decomposed on a Gabor over-complete dictionary (GD), Gaussian model over-complete dictionary (GMD), and adaptive morphological over-complete dictionary (AMCD), and then they are reconstructed by the five atoms with maximum representation coefficients. The first two are structural dictionaries, and the last one is a non-structural dictionary. The residual energy between the original image and the reconstructed image is introduced to evaluate the capability of sparse representation.

**Figure 9.**
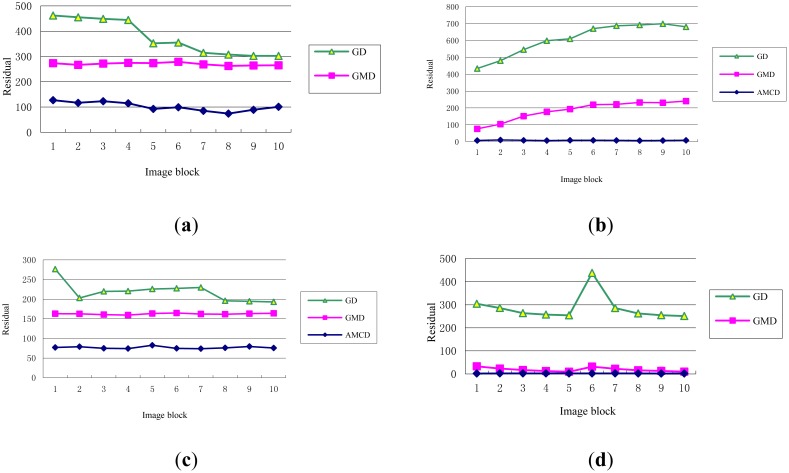
Residual energy after image reconstruction. (**a**) and (**b**) are target blocks in space image and cloud image, respectively. (**c**) and (**d**) are background blocks in space image and cloud image, respectively.

Obviously, the smaller the residual energy is, the more powerful the sparse representation would be. Ten target image blocks and ten background image blocks in space image and cloud image are decomposed and reconstructed, and their residual energy (not normalization) are shown in Figure 9. For the twenty image blocks, the residual energy of AMCD is the minimum, and that of GD is the maximum. This figure indicates that structural dictionary AMCD, which is trained according to image content, could more effectively describe the morphological component than these non-structural dictionaries GD and GMD, and its sparse representation ability is the most powerful.

**Figure 10.**
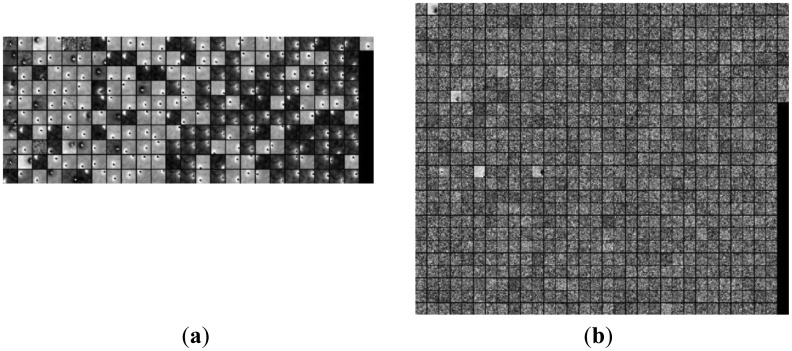

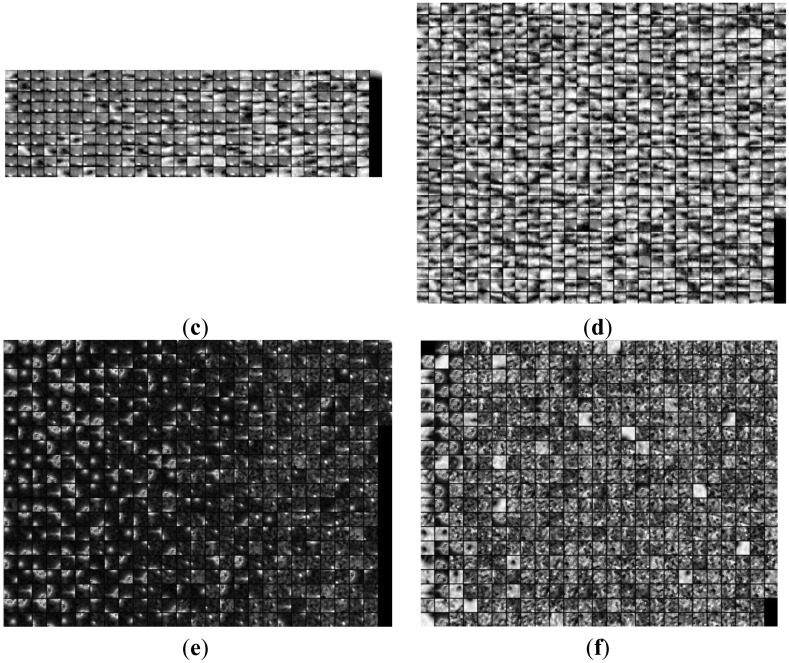
Discriminative over-complete dictionary. (**a**) and (**b**) are target dictionary and background dictionary for space image, respectively; (**c**) and (**d**) are target dictionary and background dictionary for cloud image, respectively; (**e**) and (**f**) are target dictionary and background dictionary for multi-target image, respectively.

The discriminative over-complete dictionaries for space image, cloud image and multi-target image are shown in Figure 10. Figure 10a and 10b are the target over-complete dictionary and background over-complete dictionary for space image, respectively; Figure 10c and 10d are the target over-complete dictionary and background over-complete dictionary for cloud image, respectively; Figure 10e and 10f are the target over-complete dictionary and background over-complete dictionary for multi-target images, respectively. The threshold *δ* used to distinguish target over-complete dictionary and background over-complete dictionary is equal to three multiplied by size of the atom, and the parameter *k* is equal to five. The target atoms are bright points, which are located at various positions of the image blocks with diverse shapes. The background atoms are noise and cloud clutter for space image, cloud image, and multi-target image respectively. There are 250 target atoms and 894 background atoms for deep space image, 280 target atoms and 884 background atoms for cloud image, and 526 target atom and 498 background atoms for multi-target image. For the space image, three background atoms and two target atoms are wrongly identified as target atom and background atoms, respectively; for cloud image, there are 22 background atoms and nine target atoms are wrongly classified as target atom and background atom, respectively; for multi-target image, there are seven background atoms and six target atoms are wrongly classified as target atom and background atom, respectively. The correct probabilities for space image, cloud image and multi-target image are more than 98%, 91%, and 95%, respectively, yet, depending on the complex degree of background clutter, the detection probability by the criteria based on Gaussian over-complete dictionary fluctuates.

**Figure 11.**
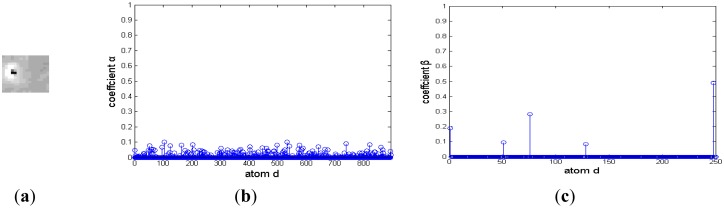
Representation coefficient of target signal in deep space image decomposed on discriminative over-complete dictionary. (**a**) Target signal. (**b**) Sparse coefficient on background dictionary. (**c**) Sparse coefficient on target dictionary.

**Figure 12.**
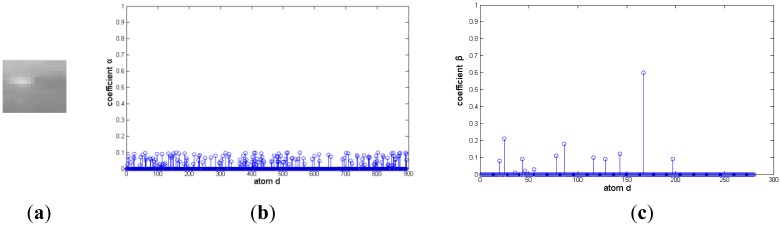
Representation coefficient of target signal in cloud image decomposed on discriminative over-complete dictionary. (**a**) Target signal. (**b**) Sparse coefficient on background dictionary. (**c**) Sparse coefficient on target dictionary.

**Figure 13.**
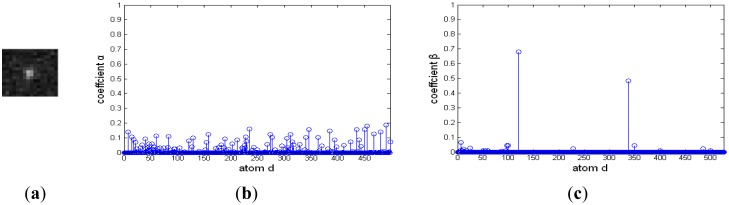
Representation coefficient of target signal in multi-target image decomposed on discriminative over-complete dictionary. (**a**) Target signal. (**b**) Sparse coefficient on background dictionary. (**c**) Sparse coefficient on target dictionary.

The representation coefficients of the target image blocks decomposed on this discriminative over-complete dictionary are shown in Figures 11, 12 and 13. In Figures 11, 12 and 13, Figures 11a– 13a are target signals, and Figures 11b–13b and Figures 11c–13c are the coefficients on corresponding background over-complete dictionary and corresponding target dictionary, respectively. There are many nonzero coefficients in Figures 11b–13b of Figures 11, 12 and 13, and this means that the signal couldn't be sparsely represented by background over-complete dictionary. In Figures 11, 12 and 13, Figures 11c–13c have less nonzero coefficients than that of Figures 11b–13b, and it indicates the target image blocks could be sparsely represented by target over-complete dictionary. Moreover, the residual reconstructed by background over-complete dictionary is much than that of target over-complete dictionary with the same *m* atoms, Here, *m* is equal to five. The target signal could be reconstructed by five target atoms with maximum nonzero coefficients in the corresponding target over-complete dictionary, and the residuals of deep space image, cloud image and multi-target image are very small, about 0.13, 0.17 and 0.09 (normalization), respectively. Otherwise, their residuals after reconstructed using five background atoms with maximum nonzero coefficients in corresponding background over-complete dictionary are very big, and they are 0.94, 0.91 and 0.89, respectively. The residuals reconstructed by corresponding target over-complete dictionary *r_t_*(*f*) are less than that constructed by corresponding background over-complete dictionary *r_b_*(*f*), and the image would be correctly labeled as target.

**Figure 14.**
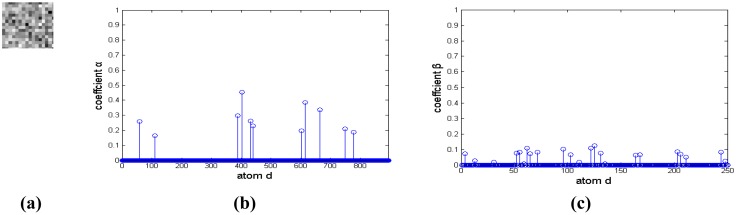
Representation coefficient of background block in deep space image decomposed on discriminative over-complete dictionary. (**a**) Sparse coefficient on background dictionary, (**b**) Sparse coefficient on target dictionary.

**Figure 15.**
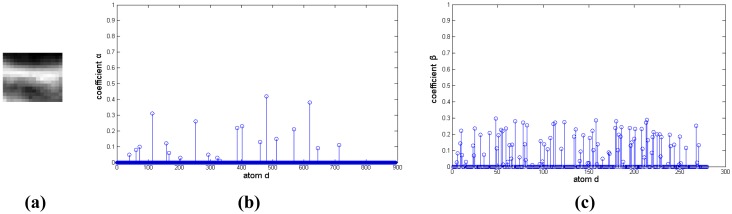
Representation coefficient of background block decomposed in cloud image on discriminative over-complete dictionary. (**a**) Sparse coefficient on background dictionary, (**b**) Sparse coefficient on target dictionary.

**Figure 16.**
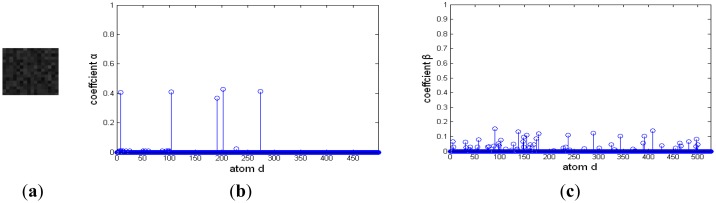
Representation coefficient of background block in multi-target image decomposed on discriminative over-complete dictionary. (**a**) Sparse coefficient on background dictionary, (**b**) Sparse coefficient on target dictionary.

The representation coefficient of the background image blocks decomposed based on this discriminative over-complete dictionary are shown in Figures 14, 15 and 16. In Figures 14, 15 and 16, Figures 14a–16a are background noise, and Figures 14b–16b and Figures 14c–16c are the representation coefficients based on the corresponding background over-complete dictionary and corresponding target over-complete dictionary, respectively. In Figures 14, 15 and 16, There are some nonzero coefficients on atoms in Figures 14b–16b and Figures 14c–16c, and Figure 14b–16b has less nonzero coefficients than that of Figures 14c–16c, and it indicates the background image blocks could be represented based on the corresponding background over-complete dictionary more sparsely than based on the corresponding target over-complete dictionary. Moreover, the residual reconstructed by the background over-complete dictionary is less than that of the target over-complete dictionary with the same atoms. The background image could be reconstructed by five background atoms with maximum nonzero coefficients in the corresponding background over-complete dictionary, and the residuals of deep space image, cloud image and multi-target image are about 0.17, 0.28 and 0.10 (normalization), respectively. The residuals reconstructed using five target atoms with maximum nonzero coefficients in corresponding target over-complete dictionary is 0.64, 0.83 and 0.75, respectively. Their residuals are *r_t_*(*f*) > *r_b_*(*f*), and the image should be labeled as background.

**Figure 17.**
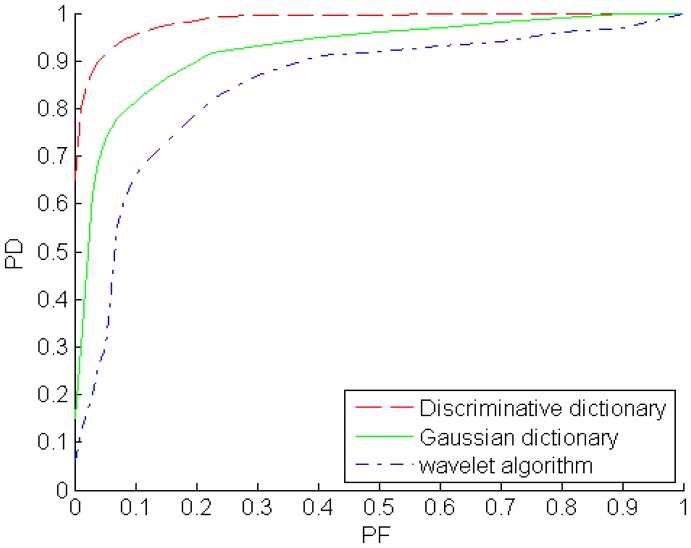
ROC curves of target detection.

The receiver operating characteristic (ROC) curves of the Gaussian over-complete dictionary, discriminative over-complete dictionary and wavelet algorithm are shown in Figure 17. The ROC curve describes the probability of detection (PD) as a function of the probability of false alarms (PF). The PF is calculated by the number of false alarms (background pixels determined as target) over the number of background samples, and the PD is the ratio of the number of hits (target pixels determined as target) and the total number of true target samples. The extent parameters $\sigma_{x}^{2}$ and $\sigma_{y}^{2}$ in the two dimensional Gaussian model are extended to some degree, background clutter, even flat background, could be represented sparsely by the Gaussian over-complete dictionary. Hence, PD and PF would be increased with the extent parameters increasing. From the ROC plots, the discriminative over-complete dictionary outperforms the Gaussian over-complete dictionary, *i.e.,* the PD of the former is larger than that of the latter when their PFs are same. Meanwhile, the wavelet algorithm is the worst one among the three algorithms for the example images.

### 6. Conclusions

This paper proposed an infrared dim target detection approach based on a sparse representation on a discriminative over-complete dictionary. This non-structural over-dictionary adaptively learns the content of infrared images online and is further divided into a target over-complete dictionary and a background over-complete dictionary automatically. The target over-complete dictionary could describe target signals, and the background over-complete dictionary would represent background clutter. This discriminative over-complete dictionary not only can capture significant features of background clutter and dim targets better than a structural over-complete dictionary, but also can efficiently strengthen the sparse feature difference between background clutter and target signals better than a discriminative over-complete dictionary learned offline and classified manually. The experimental results show that this proposed approach could effectively improve the performance of small target detection.

When SNR is lower than one, a target signal would be submerged in the strong noise, and its shape would be polluted and be not represented simply by a Gaussian model. Future work would focus on how to more effectively distinguish the target over-complete dictionary and background over-complete dictionary from adaptive morphological over-complete dictionary for diverse target signals and background clutter even in low SNR. Moreover, the computation time of the discriminative over-complete dictionary to detect target signal is about 6.4 s for a frame image, and this proposed algorithm should be optimized to decrease the computation complexity.
